# Analysis of Key Factors Associated with Response to Salvage High-Dose Methotrexate Rechallenge in Primary Central Nervous System Lymphoma with First Relapse

**DOI:** 10.3390/curroncol29090522

**Published:** 2022-09-17

**Authors:** Peng Du, Hongyi Chen, Li Shen, Xiao Liu, Xuefan Wu, Lang Chen, Aihong Cao, Daoying Geng

**Affiliations:** 1 Department of Radiology, Huashan Hospital, Fudan University, Shanghai 200040, China; 2 Department of Radiology, The Second Affiliated Hospital of Xuzhou Medical University, Xuzhou 221000, China; 3 Academy for Engineering and Technology, Fudan University, Shanghai 200433, China; 4 Department of Radiology, Jiahui International Hospital, Shanghai 200233, China; 5 School of Computer and Information Technology, Beijing Jiaotong University, Beijing 100044, China; 6 Department of Radiology, Shanghai Gamma Hospital, Shanghai 200235, China

**Keywords:** high-dose methotrexate rechallenge, primary central nervous system lymphoma, first relapse, treatment response, PFS, KPS, chemotherapy, whole brain radiotherapy

## Abstract

Background: Primary central nervous system lymphoma (PCNSL) is a rare extranodal non-Hodgkin’s lymphoma that occurs in the central nervous system. Although sensitive to chemotherapy, 35–60% of PCNSL patients still relapse within 2 years after the initial treatment. High-dose methotrexate (HD-MTX) rechallenge is generally used in recurrent PCNSL, especially for patients who have achieved a response after initial methotrexate (MTX) treatment. However, the overall remission rate (ORR) of HD-MTX rechallenge is about 70–80%. Additionally, the side effects of HD-MTX treatment endanger the health of patients and affect their quality of life. Methods: This is a retrospective study of patients with first relapse PCNSL at Huashan Hospital, Fudan University between January 2000 and November 2020. By comparing the clinical characteristics and radiological manifestations of first relapsed PCNSL patients with remission and non-remission after receiving HD-MTX rechallenge, we screened out the key factors associated with HD-MTX rechallenge treatment response, to provide some help for the selection of salvage treatment strategies for patients with recurrent PCNSL. Additionally, patients with remission after HD-MTX rechallenge were followed up to identify the factors related to progression-free survival of the second time (PFS2) (time from the first relapse to second relapse/last follow-up). The Kruskal–Wallis and Pearson chi-square tests were performed to examine the univariate association. Further, multivariable logistic regression analysis was used to study the simultaneous effect of different variables. Results: A total of 207 patients were enrolled in the study based on the inclusion criteria, including 114 patients in the remission group (RG) and 81 patients in the non-remission group (nRG), and 12 patients were judged as having a stable disease. In Kruskal–Wallis and Pearson chi-square tests, progression-free survival rates for first time (PFS1) and whether the initial treatment was combined with consolidated whole brain radiotherapy (WBRT) were related to the response to HD-MTX rechallenge treatment, which was further validated in regression analysis. Further, after univariate analysis and regression analysis, KPS was related to PFS2. Conclusions: For PCNSL patients in their first relapse, HD-MTX rechallenge may be an effective salvage treatment. PFS1 and whether initial treatment was combined with consolidation WBRT were associated with HD-MTX rechallenge treatment response. In addition, patients with higher KPS at the time of the first relapse had a longer PFS2 after HD-MTX rechallenge treatment.

## 1. Introduction

PCNSL is a rare extranodal non-Hodgkin’s lymphoma that occurs in the central nervous system [1]. Its incidence accounts for 1–3% of intracranial malignant tumors, mainly in people aged 45–70, with an average age of 60 [2]. At present, the treatment plan for PCNSL is divided into two stages: induction therapy and consolidation therapy. The induction therapy is mainly combined chemotherapy based on HD-MTX [3]. The consolidation therapy after induction remission includes WBRT and autologous hematopoietic stem cell transplantation (AHSCT), while simple surgical resection is only used as the symptomatic treatment and qualitative diagnosis of intractable brain edema caused by tumors, with no significant survival benefit for PCNSL patients [4,5].

Although PCNSL is an obvious chemosensitive tumor, which usually achieves complete response (CR) after the initial chemotherapy, 35–60% of patients experience relapse within 2 years after the initial treatment [6]. According to the literature, about 10–30% of PCNSL patients became refractory in the standard treatment based on HD-MTX [7]. At the same time, because PCNSL is particularly sensitive to radiotherapy, many scholars advocated the use of HD-MTX combined with consolidation radiotherapy to delay the recurrence of PCNSL [8]. Owing to the fact that PCNSL usually presents with multifocal and diffuse brain infiltration, WBRT is generally selected as the preferred method of radiotherapy for patients with PSNCL [9]. However, although WBRT alone can achieve a remission rate of 60%, the related delayed nerve damage caused by it, such as cognitive impairment, incontinence, and gait disorders, seriously affects the quality of life of patients after treatment, especially for patients over 60 years old [10]. Studies have shown that due to the impact of secondary neurotoxicity, the survival rates of elderly patients with PCNSL receiving HD-MTX chemotherapy combined with or without WBRT were basically the same. Therefore, WBRT is no longer routinely recommended for newly diagnosed PCNSL patients, but as a salvage treatment for tumor recurrence [11].

For relapsed/refractory (R/R) PCNSL, many institutions and organizations have proposed a variety of salvage treatment regimens, including HD-MTX rechallenge and other chemotherapy regimens, WBRT, AHSCT, targeted therapy, and immunotherapy treatment, and more than 20 treatment regimens for R/R PCNSL have been reported in some retrospective studies [12], but there is no consensus on the optimal treatment regimen. The guidelines of the European Association for Neuro-Oncology in 2015 recommend that salvage therapy for patients with relapsed PCNSL depended on age, performance status, relapse sites in the central nervous system (CNS), previous treatment, and duration of the last remission [13]. If the patient has achieved a response after initial MTX treatment, especially lasting for over 12 months, MTX rechallenge is a safe and effective strategy for relapse [14], which may be the first choice for reasonable treatment at present [15]. It has also been indicated that the ORR of HD-MTX rechallenge is about 70–80% and delayed toxic remediation treatment may be required [16,17]. In addition, the main side effects of HD-MTX treatment are renal function injury and subsequent severe myelosuppression and mucositis, which endanger the health of patients and affect their quality of life [18]. Thus, it is of great clinical significance to find out the key factors associated with response to HD-MTX rechallenge, provide guidance for formulating individualized treatment plans for patients with relapsed PCNSL, and avoid adverse reactions caused by ineffective treatment, since avoiding overtreatment of patients with poor prognoses is of the same importance as aggressive treatment of patients who may survive for years [19].

In this study, by comparing the clinical characteristics and radiological manifestations of first relapsed PCNSL patients with remission and non-remission after receiving salvage HD-MTX rechallenge, we screened out the key factors related to HD-MTX rechallenge treatment response, to provide some help for the selection of salvage treatment strategies for patients with recurrent PCNSL. Additionally, patients with remission after HD-MTX rechallenge were followed up to identify the main factors associated with PFS2.

## 2. Methods

### 2.1. Patient Cohort

This retrospective study was approved by the institutional review board of Huashan Hospital, Fudan University (KY2021-066). The requirement for written informed consent was waived. The data of patients with first relapsed PCNSL who received HD-MTX re-challenge treatment at Huashan Hospital, Fudan University between January 2000 and November 2020 were reviewed. Eligible criteria were as follows: (1) PCNSL patient age above 18 years; (2) PCNSL patient with the first relapse; (3) the initial treatment was HD-MTX based chemotherapy with CR after 3–5 courses; (4) PFS1 (time from the first CR to the first recurrence) ≥ 6 months; (5) KPS ≥ 60 at first recurrence; (6) only HD-MTX rechallenge was used at the time of the first relapse, and no other treatment was combined; (7) immunocompetent and HIV-negative; (8) definite pathological basis for the initial diagnosis of PCNSL; (9) complete MRI data before and after HD-MTX rechallenge treatment; (10) relevant clinical data and laboratory tests were complete. 

### 2.2. Clinical Information

The collected clinical information included the following two aspects: (1) Information on the patient’s initial diagnosis of PCNSL: lesion location, histopathological characteristics, whether the lesion was removed or not, and whether the initial treatment was combined with consolidation WBRT or not; (2) Information on the patient’s first relapse: age, KPS, ECOG score, PFS1, clinical symptoms, lesion location, lesion number, serum LDH level, serum β2-MG, urine β2-MG, eGFR, cerebrospinal fluid (CSF) Pandy test, CSF protein quantification, CSF Glucose, CSF chloride, and CSF white blood cell count. The follow-up deadline for this study was 1 February 2022. PFS1 was defined as the time from the first CR to the first recurrence, and distributed between 6.0 and 17.2, with a mean value of 10.845 ± 3.157. PFS2 was defined as the time from the first relapse to the second relapse/last follow-up, and distributed between 3.5 and 14.6, with a mean value of 9.008 ± 3.726. Patients with remission after HD-MTX rechallenge were followed up and divided into three groups according to PFS2 (Group1: PFS2 < 6 months; Group2: 6 months ≤ PFS2 ≤ 12 months; Group3: PFS2 > 12 months), and the differences in clinical characteristics among the three groups were compared.

### 2.3. Treatment and Response Assessment

All patients received four courses of HD-MTX rechallenge treatment at a dose of 3.5–4.5 g/m^2^, and they underwent enhanced brain MRI before and after treatment. MRI images 60 days after treatment were used to evaluate the treatment response, and MRI images every 3 months after treatment were used for follow-up. All MRI examinations were acquired using a 1.5T MRI system (SIGNA Excite HD; GE Healthcare, Milwaukee, WI, USA) before and after HD-MTX rechallenge treatment. MRI sequences included T1 weighted imaging (T1WI), contrast-enhanced-T1WI (CE-T1WI), T2 weighted imaging (T2WI) and diffusion-weighted imaging (DWI), and the total acquisition time per patient was approximately 18 min.

Treatment responses were evaluated based on the pre-treatment MRI and the follow-up MRI 60 days after treatment. According to the International Primary CNS Lymphoma Group (IPCG) response criteria [20], patients were classified into CR, unconfirmed CR (uCR), partial response (PR), progressive disease (PD), and stable disease (SD). We defined the CR, uCR, and PR as the RG, while the PD as the nRG. 

### 2.4. Image Analysis

All the MRI images were analyzed in consensus by two experienced neuro-radiologists (with 12 and 15 years of experience in central nervous system radiological diagnosis, respectively). Tumor volume, maximum diameter, edema index (EI), T1WI sequence signal (hypo-intensity, iso-intensity, and hyper-intensity), T2WI sequence signal (hypo-intensity, iso-intensity, and hyper-intensity), DWI sequence signal (hypo-intensity and hyper-intensity), and enhanced pattern (homogeneous and heterogeneous) were assessed. The EI was calculated according to the method proposed by Kim et al. [21].

### 2.5. Statistical Analysis

Statistical analysis was performed using commercially available software (IBM SPSS Statistics 26.0 for Windows, IBM Corp, Armonk, NY, USA). For continuous and discrete data, the Kruskal–Wallis test and Pearson chi-square test were performed to examine the univariate association of the response to HD-MTX rechallenge treatment and PFS2 with any clinical characteristics or radiological manifestations. Finally, to study the simultaneous effect of different variables, multivariable logistic regression analysis was used. Initially, all the variables were included but the best model was derived using stepwise logistic regression analysis. Statistical significance was set at a probability value of 0.05.

## 3. Results

### 3.1. Clinical Characteristics

A total of 207 patients were enrolled in the study based on the inclusion criteria, including 114 patients in the RG (CR = 72, uCR = 15, and PR = 27) and 81 patients in the nRG (PD = 81), and 12 patients were judged as SD. A total of 20 clinical characteristics were selected, including recurrence age, KPS, ECOG score, PFS1, with or without clinical symptoms at the time of recurrence, the consistency of the location of recurrence and initial lesion, the lesion involved deep brain or not, lesion number, serum LDH level, serum β2-MG, urine β2-MG, eGFR, CSF Pandy test, CSF protein quantification, CSF Glucose, CSF chloride, CSF white blood cell count, the lesion was removed or not at the initial treatment, the initial treatment was combined with consolidation WBRT or not, and histopathological characteristics. Demographic and baseline characteristics of clinical characteristics are shown in Table 1. The continuous type of the variable PFS1 was distributed between 6.0 and 17.2, with a mean value of 10.845 ± 3.157. Table 2 showed the summary of clinical characteristics by PFS2 grouping.

Comparing the difference in clinical characteristics between first relapse PCNSL patients with remission and non-remission after HD-MTX rechallenge, it was found that PFS1 and whether the initial treatment was combined with consolidated WBRT (*p* = 2.504 × 10^−8^ and 2.000 × 10^−6^, respectively; Table 3) were related to the response to HD-MTX rechallenge treatment. After exclusion by stepwise regression multivariate analysis, PFS1 and whether the initial treatment was combined with consolidated WBRT were found to be associated with the response to HD-MTX rechallenge treatment (Table 4). 

**Figure 1:**
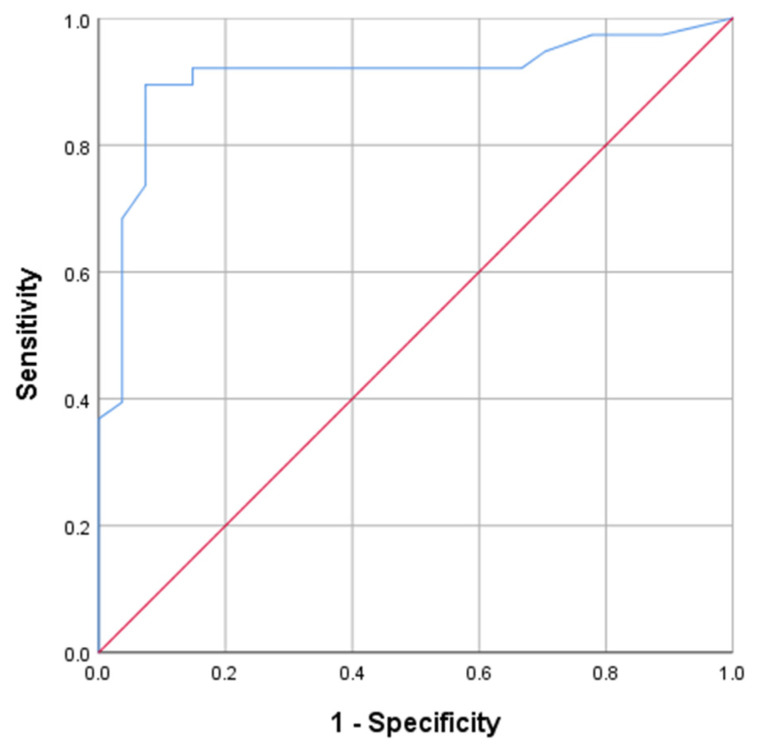
ROC curve for PFS1.

**Figure 2:**
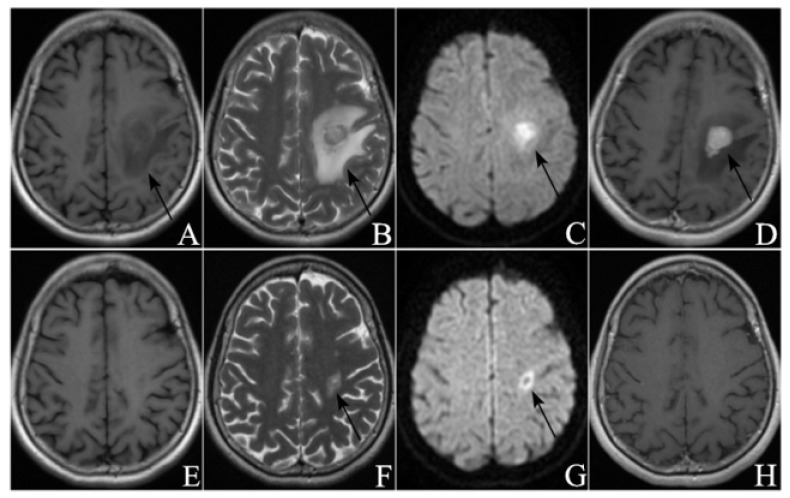
A sixty-five-year-old male PCNSL patient received consolidation WBRT after initial MTX chemotherapy, and the PFS1 was 12.3 months. KPS of this patient was 70 at first relapse. (**A**–**D**) were MRI images before HD-MTX rechallenge, and (**E**–**H**) were MRI images 60 days after treatment ((**A**,**E**) from T1WI, (**B**,**F**) from T2WI, (**C**,**G**) from DWI, (**D**,**H**) from CE-T1WI). The tumor was located in the left frontal lobe and showed homogeneous enhancement before treatment. After treatment, no abnormal enhancement appeared on CE-T1WI, and this patient was judged as CR.

**Figure 3:**
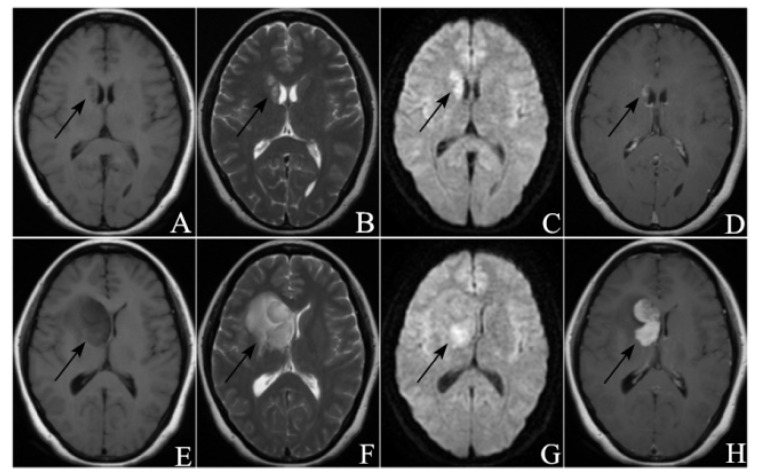
A sixty-seven-year-old male PCNSL patient didn’t receive consolidation WBRT after initial MTX chemotherapy, and the PFS1 was 11.2 months. KPS of this patient was 70 at first relapse. (**A**–**D**) were MRI images before HD-MTX rechallenge, and (**E**–**H**) were MRI images 60 days after treatment ((**A**,**E**) from T1WI, (**B**,**F**) from T2WI, (**C**,**G**) from DWI, (**D**,**H**) from CE-T1WI). The tumor was located in the right basal ganglia and showed homogeneous enhancement. After treatment, the tumor obviously enlarged and the edema aggravated, and this patient was judged as PD.

The ROC curve for PFS1 was plotted in Figure 1. The maximum value of the Youden index was calculated to be 0.821, corresponding to a cut-off value of 11.750. This means that treatment response will more likely be remission when PFS1 is more than 11.750 months. Figure 2 showed the MRI images of a patient in the remission group pre-and-post treatment, and Figure 3 showed the MRI images of a patient in the non-remission group pre-and-post treatment.

Patients with remission after the HD-MTX rechallenge were followed up and divided into three groups according to PFS2 (PFS2 < 6 months, 6 months ≤ PFS2 ≤ 12 months, and PFS2 > 12 months). By comparing the differences in clinical characteristics among the three groups, KPS (*p* = 2.000 × 10^−6^; Table 5) was found to be associated with PFS2. Multivariable logistic regression models were developed to search for independent predictors of PFS2. As the analysis showed, KPS was the only independent predictor (*p* = 3.932 × 10^−7^, Table 6).

### 3.2. Radiological Manifestations

A total of 7 radiological manifestations were selected, including tumor volume, maximum diameter, EI, T1WI sequence signal, T2WI sequence signal, DWI sequence signal, and enhanced pattern. The volume distribution was between 351.25 and 4689.72 with a mean volume of 1575.752 ± 822.986. The maximum diameter ranged from 0.530 to 4.110 with a mean value of 2.005 ± 0.998. The EI ranged from 1.040 to 4.860 with a mean value of 2.498 ± 0.917. Other baseline characteristics of radiological manifestations are listed below (Table 7).

Comparing the difference in radiological manifestations between first relapse PCNSL patients with remission and non-remission after HD-MTX rechallenge, no factors were found to be associated with response to HD-MTX rechallenge treatment (Table 8). 

Further, the differences in radiological manifestations among the three groups (PFS2 < 6 months, 6 months ≤ PFS2 ≤ 12 months, and PFS2 > 12 months) were analyzed. Univariate analysis showed that there were no significant factors associated (Table 9).

## 4. Discussion

Relapsed PCNSL patients generally have poor prognoses, with a median survival of only two months without additional treatment [22]. At present, there is no consensus on the optimal treatment regimen for recurrent PCNSL [23]. No randomized trials have been conducted so far in this patient population, in part because of (1) limited insights into the pathophysiology of this disease pointing to specific drug targets and (2) the heterogeneous sites of recurrence (brain, CSF, eyes, or a combination thereof), number of recurrences, and age at recurrence [24]. Therefore, many salvage treatment options for patients with relapsed PCNSL are based on the patient’s physical status, previous treatment methods, the wishes of patients and their families, and the clinical experience of doctors in charge, which lacks objectivity and standardization [25]. As a result, many patients have adverse reactions that seriously affect the prognosis and quality of life due to inappropriate treatment selections.

Since Ervin et al. [26] first reported that a patient with relapsed PCNSL achieved CR after HD-MTX rechallenge in 1980, MTX alone or in combination with other chemotherapeutics became the main treatment cornerstone of relapsed PCNSL [27]. In a retrospective study by Plotkin et al. [17], 22 patients with relapsed PCNSL who achieved a response with HD-MTX initial treatment received HD-MTX (≥3 g/m^2^) rechallenge as a single regimen for two salvage therapies. The ORR for the first rechallenge was 91%, and that for the second rechallenge was 100%; moreover, the median overall survival (mOS) after the first relapse was 61.9 months. A study by Pentsova et al. [16] also supported MTX-based salvage therapy for relapsed PCNSL; the ORR was 85%, the 1-year OS was 79%, and the mOS was 41 months. For patients with relapsed PCNSL, HD-MTX rechallenge may be a reasonable treatment option if a response is achieved after initial HD-MTX therapy [28]. The National Comprehensive Cancer Network (NCCN) guidelines version 3.2020 recommend adjusting treatment strategies based on the patient’s initial treatment and response duration. If the patient has achieved a response after initial MTX treatment, especially lasting for over 12 months, MTX rechallenge is a safe and effective strategy for relapse [14]. However, there still exists controversy over the definition of the minimum effective remission period for HD-MTX rechallenge therapy.

Although the ORR of HD-MTX rechallenge can reach above 70%, some patients still experience disease progression and may require delayed toxic remedial treatment [16,17]. Moreover, the main side effects of HD-MTX treatment include renal function injury, myelosuppression, and mucosal injury, which may seriously affect the prognosis and quality of life of patients [29]. Therefore, finding the key factors related to HD-MTX rechallenge treatment response may be able to prevent patients from being affected by unnecessary adverse treatment reactions to a certain extent. If the patient is found to have risk factors for non-remission after treatment, it is conducive to more effective communication between doctors, patients, and their families, to formulate individualized salvage treatment plans according to the actual situation of patients, so as to maximize the prognoses of patients.

This study demonstrated that patients who received consolidation WBRT in initial treatment were more likely to achieve remission with HD-MTX rechallenge at the first relapse, possibly due to: (1) consolidated WBRT can alleviate subclinical lesions to a certain extent, and then HD-MTX chemotherapy can further strengthen the therapeutic effect; (2) WBRT combined with HD-MTX chemotherapy can improve the sensitivity of PCNSL to treatment and improve the treatment effect; (3) radiotherapy can significantly reduce tumor volume, change the distribution of blood vessels in the tumor, and increase the sensitivity of residual tumor cells to chemotherapy drugs. For a long time, because the vast majority of drugs are difficult to penetrate the blood-brain barrier and cannot reach effective therapeutic concentrations in the brain, resulting in poor chemotherapy effect [30]. Some scholars believed that the permeability of the blood-brain barrier was significantly increased when the whole brain was irradiated with 20–30 Gy, which was the best time for chemotherapy. The research by Abrey et al. [25] indicated that the combination of MTX-based chemotherapy and WBRT could significantly improve the treatment efficiency of PCNSL (up to 94%), and the OS could be improved to 33–60 months. Ferreri et al. [23] opined that primary chemotherapy followed by radiotherapy as a therapeutic modality could improve the cure rate of PCNSL. The two-year survival was about 60–70%, and the five-year survival could be increased to 22–40%. Chanswangphuwana et al. [31] retrospectively analyzed the effect of WBRT as consolidation therapy in patients with PCNSL relapse, and some of the 37 patients with newly diagnosed PCNSL underwent WBRT after initial remission. The results showed that among the 22 patients with CR, progression-free survival (PFS) was significantly lower in patients without WBRT than in patients with WBRT, and the 3-year PFS rates were 35% and 78.75%, respectively. However, some studies have shown that the mPFS of PCNSL patients who received combined therapy was significantly prolonged, but the mOS was not improved, which may be related to the adverse neurological reactions caused by WBRT [32]. Thus, Hoang-Xuan et al. [13] recommended salvage radiotherapy as a treatment option for young patients with R/R PCNSL. Some scholars believed that low-dose WBRT was an effective treatment for PCNSL [33]. Thus, initial consolidation therapy may play an important role in relapsed PCNSL [34].

Gisselbrecht et al. [35] reviewed the clinical information of 396 patients with CD20 (+) diffuse large B-cell lymphoma (DLBCL) in first relapse or who were refractory after first-line therapy and found that PFS1 was an important prognostic factor for R/R DLBCL. Ferreri et al. [36] retrospectively analyzed the treatment-related variables in a multicenter series of 378 PCNSL patients treated at 23 cancer centers in five different countries, and it was indicated that PFS1 was of great significance in guiding the treatment of R/R PCNSL. Langner-Lemercier et al. [37] conducted a retrospective study on the clinical data of 256 patients with R/R PCNSL and concluded that PFS1 < 1 year was an independent risk factor affecting the prognosis of first R/R patients. This study found that patients with PFS1 for more than 12 months were more likely to achieve remission with HD-MTX rechallenge at the time of the first relapse, which was basically consistent with the conclusions of previous literature. For PCNSL patients, longer PFS1 may mean that the initial treatment was more effective in controlling the disease, and the repeated use of the initial treatment regimen in the event of relapse may achieve satisfactory results. However, it is still necessary to carry out a study with a larger sample size in the future to verify this inference.

KPS is the evaluation standard for the quality of life of patients with cancer, which was proposed by Karnofsky of the Eastern Cooperative Oncology Group (ECOG) and originally used to evaluate whether patients with cancer can tolerate chemotherapy [38]. KPS is based on the patient’s condition, normal activity, and self-care. The higher the score is, the better the patient’s health will be. At present, KPS is also widely used in the evaluation of patients with cancer before receiving other treatments, and patients with higher KPS are more able to tolerate the side effects caused by treatment [39]. Nelson et al. [40] believed that a low KPS value was a strong negative predictor of outcome for non-Hodgkin’s lymphoma of the brain. The research by Abrey et al. [41] indicated that PCNSL patients aged >50 years with a KPS of <70 had the worst prognosis, with a median survival of 1.1 years. Houillier et al. [42] opined that KPS at diagnosis ≥ 70 and response to initial chemotherapy were associated with increased OS in PCNSL patients. Additionally, Pentsova et al. [16] believed that KPS was a prognostic factor for PFS of recurrent PCNSL. This study showed that patients with KPS > 70 at the time of the first relapse had a longer PFS2 after HD-MTX rechallenge treatment, which was similar to the conclusions of the above literature.

The radiological manifestations of PCNSL are closely related to its pathological characteristics. The tumor forms a “sleeve-like” structure centered on the perivascular space, and most of the multiple lesions spread through the perivascular space, so they are relatively limited to local brain tissue [43]. The density and signal of PCNSL are generally uniform, and necrosis, cystic degeneration, calcification, and hemorrhage within the tumor are rare. None-contrast CT often shows equal or slightly high density. MRI T1WI often shows iso-intensity or hypo-intensity, and T2WI mostly shows iso-intensity or hyper-intensity. DWI generally shows hyper-intensity because the diffusion of water molecules is limited due to the dense tumor cells [44]. PCNSL is a kind of hypovascular tumor, but the tumor infiltrates outward with the perivascular space as the center, invades the adjacent brain parenchyma and even the vascular wall, and destroys the blood-brain barrier. Therefore, no flow void vascular shadow can be seen in the lesion on unenhanced scans, but most of them show obvious uniform enhancement on enhanced scans. Typical cases will show a “notch sign” and a “sharp angle sign”. When the tumor involves the corpus callosum, the “butterfly sign” can be seen on the coronal MRI [45]. Unlike glioma, the peritumoral edema of most PCNSL is disproportionate to the tumor volume. The peritumoral edema is generally mild to moderate, and the edema will also spread along the perivascular space with the tumor cells [46]. This study did not find a correlation between the radiological manifestations of PCNSL and HD-MTX rechallenge treatment response. Additionally, no relevance was found between the radiological manifestations of PCNSL and PFS2 in first relapse patients with remission after HD-MTX rechallenge.

The study of Ferreri et al. [36] indicated that the factors affecting the prognosis of PCNSL patients included: (1) age ≥ 60 years; (2) ECOG score 2–4; (3) tumor involving deep brain tissue; (4) serum LDH level increased; (5) CSF protein content increased. Based on this investigation, they established the International Extranodal Lymphoma Study Group (IELSG) score system. Abery et al. [41] believed that the main factors affecting the prognosis of PCNSL were age and performance status. The research by Ahn et al. [47] pointed out that older age, multiple lesions, and increased CSF protein content were related to the prognosis of PCNSL. Some scholars also opined that the indicators of blood biochemical examination and immunological examination were also related to the prognosis of PCNSL. The serum LDH level was an independent prognostic factor of PCNSL, and patients with significantly higher indicators have poorer prognoses [36,48]. This study found that age, various prognostic score systems, tumor location, CSF protein content, blood biochemical examination, and immunological examination indicators were not associated with HD-MTX rechallenge treatment response, nor was there any correlation between the above factors and PFS2 in first relapse patients with remission after HD-MTX rechallenge.

However, our study still has some inevitable limitations. First, this was a retrospective study and not a randomized trial, lending to its inherent limitations. Secondly, magnetic resonance spectroscopy (MRS) and perfusion weighted imaging (PWI) sequences can provide more valuable information and play important roles in the diagnosis of brain tumors, which were not included in this study, and therefore we need to add them to future studies. Moreover, multi-centered, prospective, and randomized controlled clinical research on the treatment response of patients with PCNSL to HD-MTX rechallenge requires to be carried out.

## 5. Conclusions

For PCNSL patients in their first relapse, HD-MTX rechallenge may be an effective salvage treatment. PFS1 and whether initial treatment was combined with consolidation WBRT are associated with HD-MTX rechallenge treatment response. If patients are found to have risk factors for non-remission after treatment, it is conducive to more effective communication between doctors, patients, and their families, and to the formulation of individualized salvage treatment plans according to the actual situation of patients, so as to maximize the prognoses of patients. Moreover, patients with higher KPS at the time of the first relapse had a longer PFS2 after HD-MTX rechallenge treatment.

## Figures and Tables

**Table 1:** Summary of clinical characteristics.

Variable	No. of Patients (%)
Age	
Age ≥ 60	165 (84.6)
Age < 60	30 (15.4)
KPS	
KPS ≥ 70	132 (67.7)
KPS < 70	63 (32.3)
ECOG	
0	12 (6.2)
1	120 (61.5)
2	63 (32.3)
With clinical symptoms	
no	60 (30.8)
yes	135 (69.2)
Location of recurrence and initial lesion	
consistent	69 (35.4)
inconsisitent	126 (64.6)
Deep brain involved	
no	129 (66.2)
yes	66 (33.8)
Number	
single	93 (47.7)
multiple	102 (52.3)
Serum LDH	
normal	132 (67.7)
abnormal increase	63 (32.3)
Serum β2-MG	
normal	135 (69.2)
abnormal increase	60 (30.8)
Urine β2-MG	
normal	123 (63.1)
abnormal increase	72 (36.9)
eGFR	
normal	147 (75.4)
abnormal	48 (24.6)
CSF Pandy test	
positive	96 (49.2)
negative	99 (50.8)
CSF Protein quantification	
normal	78 (40.0)
abnormal increase	117 (60.0)
CSF Glucose	
normal	126 (64.6)
abnormal	69 (35.4)
CSF Chloride	
normal	117 (60.0)
abnormal	78 (40.0)
CSF White blood cell count	
normal	129 (66.2)
abnormal increase	66 (33.8)
Lesion removed at the initial treatment	
yes	63 (32.3)
no	132 (67.7)
WBRT after initial treatment	
yes	84 (43.1)
no	111 (56.9)
Histopathological characteristics	
DLBCL-non-GCB	120 (61.5)
DLBCL-GCB	69 (35.4)
DLBCL-unknown	6 (3.1)

**Table 2:** Summary of clinical characteristics by PFS2 grouping.

Variable	PFS2 < 6 Months (n = 39)	6 Months ≤ PFS2 ≤ 12 Months (n = 39)	PFS2 > 12 Months (n = 36)
Age	66.770 ± 6.559	68.690 ± 6.550	67.500 ± 8.130
KPS	70.770 ± 9.541	73.080 ± 9.473	69.17 ± 9.003
ECOG (0/1/2)	3/24/12	3/27/9	0/21/15
PFS1	12.392 ± 2.845	12.685 ± 2.5816	13.200 ± 0.9195
Clinical symptoms (no/yes)	9/30	15/24	9/27
Location of recurrence (consistent/inconsisitent)	12/27	18/21	6/30
Deep brain involved (no/yes)	30/9	24/15	24/12
Number (single/multiple)	18/21	21/18	27/9
Serum LDH (normal/abnormal increase)	27/12	21/18	27/9
Serum β2-MG (normal/abnormal increase)	33/6	24/15	33/3
Urine β2-MG (normal/abnormal increase)	27/12	27/12	24/12
eGFR (normal/abnormal)	27/12	33/6	27/9
CSF Pandy test (positive/negative)	24/15	18/21	15/21
CSF Protein quantification (normal/abnormal increase)	21/18	9/30	15/21
CSF Glucose (normal/abnormal)	21/18	27/12	24/12
CSF Chloride (normal/abnormal)	30/9	21/18	18/18
CSF White blood cell count (normal/abnormal increase)	27/12	24/15	24/12
Lesion removed at the initial treatment (yes/no)	12/27	15/24	12/24
WBRT after initial treatment (yes/no)	30/9	30/9	33/3
Histopathological characteristics (DLBCL-non-GCB/ DLBCL-GCB/ DLBCL-unknown)	24/15/0	24/12/3	21/15/0

**Table 3:** Univariate analysis of the response to HD-MTX rechallenge treatment with clinical characteristics.

Variable	χ^2^	*p* Value
Age	0.348	0.555
KPS	0.022	0.882
ECOG	0.172	0.918
PFS1	-	2.504 × 10^−8^
Clinical symptoms	0.143	0.706
Location of recurrence	0.579	0.447
Deep brain involved	0.210	0.647
Number	1.145	0.285
Serum LDH	0.151	0.697
Serum β2-MG	2.499	0.144
Urine β2-MG	1.122	0.290
eGFR	0.043	0.836
CSF Pandy test	0.022	0.883
CSF Protein quantification	0.011	0.918
CSF Glucose	0.085	0.771
CSF Chloride	0.011	0.918
CSF White blood cell count	0.005	0.941
Lesion removed at the initial treatment	0.151	0.697
WBRT after initial treatment	22.680	2.000 × 10^−6^
Histopathological characteristics	0.129	0.937

**Table 4:** Regression analysis of the response to HD-MTX rechallenge treatment with significant clinical characteristics.

Variable	B	SE	*p* Value
PFS1	0.089	0.014	5.261 × 10^−8^
WBRT after initial treatment	0.317	0.091	0.001

**Table 5:** Univariate analysis of PFS2 with clinical characteristics.

Variable	χ^2^	*p* Value
Age	2.152	0.341
KPS	26.099	2.000 × 10^−6^
ECOG	1.736	0.784
PFS1	-	0.605
Clinical symptoms	0.881	0.644
Location of recurrence	2.517	0.284
Deep brain involved	0.737	0.692
Number	2.263	0.323
Serum LDH	1.345	0.511
Serum β2-MG	3.790	0.150
Urine β2-MG	0.025	0.988
eGFR	0.868	0.648
CSF Pandy test	1.103	0.576
CSF Protein quantification	2.611	0.271
CSF Glucose	0.754	0.686
CSF Chloride	2.262	0.323
CSF White blood cell count	0.177	0.915
Lesion removed at the initial treatment	0.177	0.915
WBRT after initial treatment	1.188	0.552
Histopathological characteristics	2.152	0.708

**Table 6:** Regression analysis of PFS2 with significant clinical characteristics.

Variable	B	SE	*p* Value
KPS	1.321	0.214	3.932 × 10^−7^

**Table 7:** Summary of radiological manifestations.

Variable	No. of Patients (%)
T1WI	
hypo	96 (49.2)
iso	90 (46.2)
hyper	9 (4.6)
T2WI	
hypo	6 (3.1)
iso	84 (43.1)
hyper	105 (53.8)
DWI	
hypo	6 (3.1)
hyper	189 (96.9)
Enhanced pattern	
homogeneous	171 (87.7)
heterogeneous	24 (12.3)

**Table 8:** Univariate analysis of the response to HD-MTX rechallenge treatment with radiological manifestations.

Variable	χ^2^	*p* Value
Volume	-	0.836
Maximum diameter	-	0.905
EI	-	0.714
T1WI	0.185	0.883
T2WI	0.480	0.883
DWI	0.667	0.805
Enhanced pattern	0.146	0.604

**Table 9:** Univariate analysis of PFS2 with radiological manifestations.

Variable	χ^2^	*p* Value
Volume	-	0.200
Maximum diameter	-	0.093
EI	-	0.581
T1WI	0.154	0.926
T2WI	0.154	0.926
DWI	2.225	0.329
Enhanced pattern	3.765	0.152

## Data Availability

The data presented in this study are available on request from the corresponding author. The data are not publicly available due to protecting patient privacy.
